# Reablement in Residential Aged Care (Re-RAC): study protocol for a multi-center pragmatic randomized controlled open-label trial

**DOI:** 10.1186/s13063-025-08999-0

**Published:** 2025-08-18

**Authors:** Anna Philipson, Mialinn Arvidsson Lindvall, Cecilia Pettersson, Lena Strålman, Kajsa Lidström Holmqvist

**Affiliations:** 1 Faculty of Medicine and Health, University Health Care Research Center, Örebro University, S-Huset, Vån 2, Örebro, 701 85 Sweden; 2 School of Health, Care and Social Welfare, Mälardalen University, Box 883, 721 23 Västerås, Sweden; 3 Faculty of Medicine and Health, Faculty of Health Sciences, Kristianstad University, 291 88 Kristianstad, Sweden; 4 Faculty of Medicine and Health, School of Health Sciences, Örebro University, Örebro, 701 82 Sweden; 5 Department of Neurology and Rehabilitation Medicine, Faculty of Medicine and Health, University Health Care Research Centerand, Örebro University, S-Huset, Vån 2, Örebro, 701 85 Sweden

**Keywords:** Functional status, Rehabilitation, Aged, Participation, Psychological well-being, Quality of life, Nursing homes, Patient-centered care, Primary healthcare

## Abstract

**Background:**

Living in residential aged care (RAC) facilities can be passivating and negatively impact residents’ well-being and quality of life. With a growing global population of older adults and an increasing number residing in RAC facilities, it is crucial to address these concerns. Person-centered reablement, which enhances activity and participation through tailored, multidisciplinary strategies, has shown promising results in home settings. However, its implementation in RAC facilities, especially in Sweden, requires further evaluation.

This research project will examine whether the reablement intervention in RAC (Re-RAC) impacts activity performance and satisfaction, participation, quality of life, and well-being, in older adults living in RAC facilities. Additionally, the project will evaluate the health-economic effects of the intervention and explore potential associations with the outcomes. A further aim is to describe the experiences of both the participating older adults and RAC facility staff involved in Re-RAC.

**Methods:**

This is a multi-center prospective pragmatic randomized controlled trial has two parallel groups comparing the Re-RAC intervention with usual care. A total of 86 participants are planned to be enrolled. The 8-week intervention will be evaluated using quantitative, qualitative, and health-economic methods. Data will be collected at baseline and after the intervention. Health-economic data will also be gathered 3 months before and after intervention. Primary outcomes are activity performance and satisfaction with performance captured using the Canadian Occupational Performance Measure; secondary outcomes, i.e., health-related quality of life, psychological well-being, and physical activity levels will also be evaluated. Experiences of participants and staff will be captured through individual and focus-group interviews. Cost-effectiveness will be estimated by calculating the cost per quality-adjusted life year gained.

Quantitative data will be analyzed using descriptive and comparative statistics; qualitative data will be analyzed using thematic analysis and focus-group methodology.

**Discussion:**

This study evaluates the Re-RAC intervention for older adults in RAC through a real-world pragmatic trial, examining activity performance, satisfaction, quality of life, well-being, and health outcomes for older adults in RAC facilities. The study also explores participant and staff experiences and evaluates cost-effectiveness. The results will offer valuable insights informing the future implementation and assessment of reablement interventions in RAC settings.

**Trial registration:**

ClinicalTrials.gov, ID: NCT06793501. Registered on 20 January 2025.

## Administrative information

Note: the numbers in curly brackets in this protocol refer to SPIRIT checklist item numbers. The order of the items has been modified to group similar items (see http://www.equator-network.org/reporting-guidelines/spirit-2013-statement-defining-standard-protocol-items-for-clinical-trials/).

**Table:** 

Title {1}	Reablement in Residential Aged Care (Re-RAC): study protocol for a multi-center pragmatic randomized controlled open-label trial
Trial registration {2a and 2b}	Trial registration: ClinicalTrials.gov, ID: NCT06793501. Registered on 20 January 2025. https://clinicaltrials.gov/study/NCT06793501?term=NCT06793501&rank=1.
Protocol version {3}	Version 1 – May 16, 2025
Funding {4}	Open access funding was provided by Örebro University. The Re-RAC research project was supported by Research Grants through Special Initiatives: Project Funding for Employees in Municipal Care and Elderly Care within Örebro County, grant numbers OLL-980225, OLL-1000848, OLL-1014758, and OLL-1022263. LS received salary funding co-financed by Region Örebro County and the municipalities of Örebro County. The funders have no role in the design or implementation of the study; in the collection, management, analysis, or interpretation of the data; in the preparation, review, or approval of the manuscript; or in the decision to submit the manuscript for publication.
Author details {5a}	^1^University Health Care Research Center, Faculty of Medicine and Health, Örebro University, Sweden ^2^School of Health, Care, and Social Welfare, Mälardalen University, Västerås, Sweden ^3^Faculty of Medicine and Health, Faculty of Health Sciences, Kristianstad University, Kristianstad, Sweden ^4^Faculty of Medicine and Health, School of Health Sciences, Örebro University, Örebro, Sweden ^5^University Health Care Research Center and Department of Neurology and Rehabilitation Medicine, Faculty of Medicine and Health, Örebro University, Örebro, Sweden
Name and contact information for the trial sponsor* {5b}*	Örebro University Faculty of Medicine and Health, School of Health Sciences, Örebro University, 701 82 Örebro, Sweden; Region Örebro County, Research and Education, PO Box 1613, SE-70116 Örebro, Sweden
Role of sponsor* {5c}*	The funders have no role in the design or implementation of the study; in the collection, management, analysis, or interpretation of the data; in the preparation, review, or approval of the manuscript; or in the decision to submit the manuscript for publication.

## Introduction

### Background and rationale {6a}

The proportion of older adults is growing globally [1, 2]. This demographic shift presents significant challenges in sustaining good health and providing adequate social care for older adults and the number of individuals living in residential aged care (RAC) facilities is also expected to increase [1–3]. Both the World Health Organization (WHO) [4] and national regulations, such as Sweden’s [5], affirm that older adults have the right to live a dignified life while maintaining healthy habits, and to experience active aging and well-being. Care for older adults must strive to achieve these objectives. In contrast to these intentions, studies have shown that living in RAC facilities often has a passivating effect [6–8], indicating a need for interventions to address this issue.

In Swedish RAC facilities (e.g., long-term care facilities or nursing homes), residents typically have their own apartments and share common areas. Staff are available around the clock, primarily managing household duties. RAC facilities often provide group activities and opportunities for residents to move around within the building, fostering interaction in shared spaces (e.g., during food preparation) or in outdoor areas [9]. However, despite this adapted and accessible environment, older adults living in RAC facilities often struggle to influence their daily lives, participate in activities, and act spontaneously to the same extent as before. Consequently, their personal priorities risk becoming secondary to staff routines [6], resulting in passivity.

This passivity can lead to negative health outcomes and diminished quality of life (QoL) [7, 8, 10–13]. Additionally, depressive symptoms, feelings of loneliness, and social withdrawal are common among older adults in RAC facilities [7, 14, 15].

Self-determination, autonomy, and a sense of freedom [16, 17], along with the ability to engage in or perform meaningful activities, are closely linked to high QoL [18]. However, institutional settings such as RAC facilities can negatively affect older adults’ perceptions of their QoL [10]. Therefore, it is essential to adopt approaches that counteract passivity and strive to maintain a daily life that includes actions for autonomy and opportunities to practice meaningful activities. Unfortunately, rehabilitation resources designed to enhance the performance of meaningful activities in RAC facilities are often limited, leading to inequitable access to rehabilitation services based on residents’ needs [19]. One approach with potential to promote a more active life in RAC facilities is reablement [20].

Reablement is an approach intended to enhance an individual’s physical and functional abilities, with the goal of increasing or maintaining their level of self-reliance in meaningful daily activities and reducing their reliance on long-term services [21]. The core principles of reablement are person-centeredness and multidisciplinarity. The approach involves an initial comprehensive assessment of activity performance, followed by regular reassessments and the development of a goal-oriented support plan tailored to each individual [21]. Reablement has been shown to significantly improve daily functioning, motivation, engagement, and progress toward a reduced dependence on others. Additionally, it has demonstrated positive effects on the caregiving skills and well-being of carers [22–24].

Previous research on reablement has primarily focused on older adults living in ordinary housing with the aim of extending individuals’ independence and decreasing their reliance on care [25–27]. However, a literature review of reablement in RAC facilities suggests that this approach may hold promise in RAC settings but needs further exploration and adaptation to this specific context [20]. Not least to also evaluate the potential benefits against the possible risks of participating in reablement for this population.

In Sweden, reablement interventions in RAC facilities have not previously been studied. To address this gap, our research group has developed a reablement intervention tailored to the Swedish context, referred to as Reablement in Residential Aged Care (Re-RAC). A feasibility study was conducted to assess the intervention and study procedures, demonstrating their practicality and applicability [28]. The evaluation revealed no significant harms, which was anticipated given that the program is designed to enhance functional ability through use of low-intensity, individualized activities adapted to each participant’s capacity. Nonetheless, potential risks that may include temporary fatigue, frustration, or reduced motivation require continued investigation and evaluation on a larger scale. Thus, further evaluation is necessary through a larger-scale study using a randomized controlled trial (RCT) design to establish its efficacy and generalizability.

### Objectives {7}

The Re-RAC research project will, in a pragmatic randomized controlled trial (PrRCT), examine whether the Re-RAC intervention impacts activity performance and satisfaction, participation, quality of life, and well-being, in older adults living in RAC facilities. Additionally, the project aims to evaluate the health-economic effects of the intervention and explore potential associations with the outcomes. A further aim is to describe the experiences of both the participating older adults and the RAC facility staff involved in Re-RAC.

### Trial design {8}

**Fig. 1:**
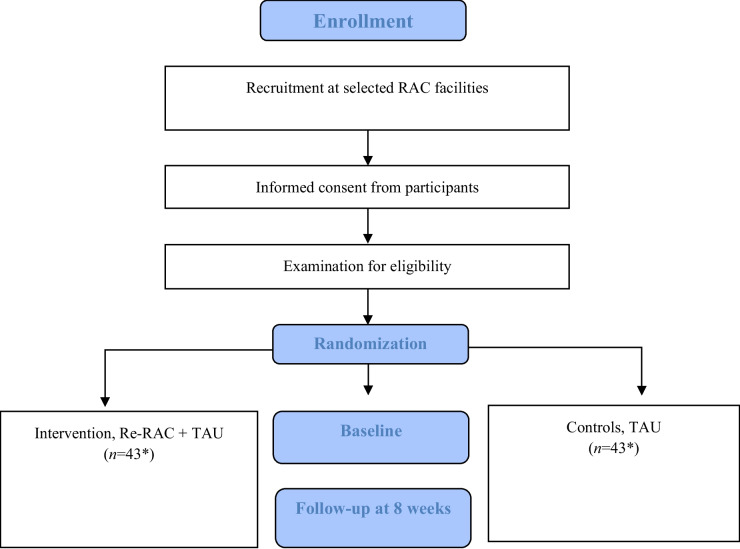
Re-RAC flowchart Abbreviations: RAC = residential aged care; Re-RAC = Reablement in RAC, TAU = treatment as usual * According to sample size calculation

This study is a multi-center prospective PrRCT with two parallel arms that will evaluate the Re-RAC intervention compared with treatment as usual (TAU) using quantitative, qualitative, and health-economic methods. The flow diagram of the study is presented in Fig. 1.

The study will be conducted as a superiority trial, evaluating differences in treatment outcomes between the two groups, randomized in a 1:1 ratio. Additionally, an incremental cost–utility analysis will be performed to evaluate the cost-effectiveness of the intervention. Individual interviews analyzed using qualitative thematic analysis [29] will be conducted to explore the older adults’ experiences of the intervention, while analysis of focus-group interviews with staff will follow the methods described by Kreuger and Casey [30] and Dahlin-Ivanoff and Holmgren [31].

This protocol follows the Standard Protocol Items: Recommendations for Interventional Trials (SPIRIT) guidelines [32]. The study will be reported in several papers according to the Consolidated Standards of Reporting Trials (CONSORT) guidelines [33], the Consolidated Criteria for Reporting Qualitative Research (COREQ) [34], and the Consolidated Health Economic Evaluation Reporting Standards (CHEERS) [35].

## Methods: participants, interventions and outcomes

### Study setting {9}

In Sweden, older adults who require extensive care that cannot be provided in ordinary housing can apply for an apartment in an RAC facility. A social assistance officer assesses and approves allocation to a RAC facility.

This study will be conducted in six RAC facilities—Rynningeviken, Ängen, Kornellen, Jeremiasgården, and two facilities still being recruited—located in Örebro municipality with about 160,000 inhabitants in central Sweden. These RAC facilities consist of several wards, each specializing in one of two areas: somatic or dementia care. Each facility has a minimum of 56 single-room apartments, with eight to ten residents per ward. The number of wards with somatic specialization varies between two and eight. The facilities also have common areas for dining and social activities. The RAC facilities provide social care and healthcare around the clock and have access to occupational therapists (OT) and physiotherapists (PT) when needed. The OTs are stationed at each RAC facility, whereas the PTs are commonly stationed at a nearby primary healthcare center and make regular visits to the RAC facility.

### Eligibility criteria {10}

RAC facilities in the specific municipality having wards with somatic specialization are eligible for the research project. The RAC facilities should not be involved in other rehabilitation research projects at the time, and they should have a stable staffing situation.

The staff supporting the older adults in the Re-RAC intervention will include nursing assistants, registered nurses, PTs, and OTs. They must be regularly employed or, in the case of external employment, be regular consultants at the RAC facilities and have completed the Re-RAC training.

Older adults aged 65 years and above, residing in a somatic ward at a RAC facility where the study will be conducted and meeting the inclusion criteria, are eligible to participate in the study. The inclusion criteria require that the older adult must be able to engage in conversations in Swedish and be able to participate in both the assessments and the intervention. Persons having severe cognitive impairment (≤ 17) [36] according to Mini Mental State Examination—Swedish version (MMSE-SR; screening for cognitive function [37]) or being very severely frail or terminally ill according to the Clinical Frailty Scale (≥ 8) (CFS-9, Swedish version [38]) will be excluded. The psychometric features for the MMSE-SR and CFS-9 are reportedly good [36, 38].

Personnel, irrespective of profession, who have completed the Re-RAC training, have worked with the intervention with at least one participant, and are Swedish speaking are eligible to participate as staff informants in the focus-group interviews.

### Who will take informed consent? {26a}

The overall management of RAC facilities in the specific municipality will sign a funding agreement, obtained by the Principal Investigator (PI). A member of the research team will meet potential participants—older adults living in an RAC facility selected for the study—who will provide written informed consent prior to the eligibility screening. Staff members who wish to participate as informants in the focus group interviews will also provide written consent to the researcher.

Participants and staff informants will be informed that they, without giving any reason, can withdraw from participation in the study at any time.

As per our exclusion criteria, individuals who are assessed to lack the capacity to provide informed consent at the time of recruitment will not be eligible to participate in the study. The current study does not include provisions for participation via legally authorized representatives.

### Additional consent provisions for collection and use of participant data and biological specimens {26b}

Data on healthcare consumption, pharmaceutical prescriptions, adverse events (AE), and adherence to the intervention will be extracted from medical records and healthcare administration systems. In the consent form, participants will be requested to allow the extraction of such data.

This study does not involve the collection and storage of biological samples.

## Interventions

### Explanation for the choice of comparators {6b}

In this study, Re-RAC will be compared with standard treatment as usual (TAU). TAU was chosen as the comparator because this study will be conducted in a real-world context in which, for ethical reasons, we cannot withhold standard treatment from any participant. Instead, Re-RAC will be an add-on intervention to TAU. The intervention group will receive both TAU and the Re-RAC intervention for 8 weeks, while the control group will receive only TAU.

The control group will be offered the Re-RAC intervention 3 months after the intervention has ended.

### Intervention description {11a}

#### Treatment as usual

TAU comprises a general approach in which all activities that the older adult engages in during everyday life are considered rehabilitative. The staff are encouraged to motivate the older adult to do as much as possible independently, to prevent loss of function and maintain the ability to perform activities. This approach does not involve any new direction toward specific activities or formulated personal goals. It does not include any formulated goals or follow-up. Additionally, no specific support is offered to the nursing assistants on how to encourage the older adult or how to follow up on the extent to which the approach is applied.

#### The Intervention: Reablement in Residential Aged Care (Re-RAC)

The Re-RAC intervention is described according to the TIDieR checklist and guide [39]. The checklist is available from the corresponding author on request.

##### Theoretical foundation

Re-RAC is rooted in the concept of reablement [21], which, in the field of aged care, is frequently used to denote a goal-oriented approach aimed at promoting meaningful activity [12, 40]. To further address this in Re-RAC, the Canadian Model of Occupational Performance and Engagement (CMOP-E) has been applied as a theoretical framework, emphasizing that engagement in activities involves motivation and willingness, and is based on what the individual finds meaningful [41].

The implementation of reablement in this study is characterized by a comprehensive assessment of the older person’s everyday activities, both current and past. This is done through a person-centered interview using the Canadian Occupational Performance Measure (COPM) [42, 43]. The COPM is based on the assumptions described in the theoretical framework of CMOP-E [41]. Based on the assessment, the older adult selects a personal activity goal, which consists of an activity they consider meaningful and wish to improve. Through collaboration between the older adult and staff, a goal-oriented support plan is developed to achieve the goal. Nursing assistant staff support the older persons in performing defined training activities and receive supervision from rehabilitation staff. The support plan and progress toward the activity goal are evaluated through regular reassessments during a fixed intervention period of 8 weeks. Hence, RE-RAC is based on the reablement concept, with multiple visits and support delivered by an educated and coordinated interdisciplinary team [21].

##### Materials and procedures

The following materials will be distributed to the nursing assistants, registered nurses, OTs, and PTs working at the participating RAC facilities:

- Educational material for a half-day training session on the Re-RAC intervention, including handouts of the presentation.
- All study materials that staff will use during the trial will be distributed and explained during the training session. These include a logbook for documenting intervention activities, instructions for using the accelerometers, the individual support plan template, and instructions for conducting team meetings.

All potential study participants will receive:

- Written information about the study, the procedure for participation, potential risks, voluntariness, and data management, as well as a consent form.

All included study participants will receive:

- An information sheet that can be distributed to the participants’ relatives; and
- Data collection material (e.g., instruction for accelerometers).

All participants in the intervention group will receive:

- A person-centered, goal-oriented support plan that includes their goal and a schedule outlining the activities for each week;
- If the training involves any assistive devices, these will be prescribed and introduced jointly by an OT or PT and the nursing assistants; and
- A logbook placed in each intervention participant’s apartment for staff to complete.

##### Preparatory training and support for staff before and during intervention

Before the start of the study, all staff at the included wards will participate in a half-day (3.5-h) training session covering both theory and practice. Topics will include reablement, gerontology, motivational strategies, and teamwork. Case scenarios based on person-centeredness, healthy aging, and previous research on reablement will be presented and discussed in smaller groups. The training will also provide information about the practical implementation of Re-RAC, including motivational strategies and empathetic engagement. A film featuring a voluntary participant in the feasibility study [28] will be used to illustrate the process, from the initial interview to the implementation of the goal-oriented support plan and teamwork, further clarifying the Re-RAC working method.

The rehabilitation staff at each participating RAC facility will also participate in an additional half-day (3.5-h) training session specifically focused on helping nursing assistants in the implementation of the support plan and conducting check-ups. Check-ups will be offered in weeks 3 and 6 by the OT to provide support to both the participants and the nursing assistants. These check-ups are intended to identify any necessary adjustments to the intervention’s content and to provide ongoing support to both the participant and the nursing assistants as they work through Re-RAC. An additional purpose of these check-ups is to help the nursing assistants maintain the participants’ motivation and engagement throughout the process.

During the intervention, regular team meetings (involving nursing assistants, nurses, and the OT) held every other week and ward meetings (including all ward staff and the ward manager) held every fourth week will be used to communicate information about the intervention and the specific activities for each participant. These forums will also provide opportunities for reflection and guidance among the staff. Additionally, the OT will offer individual oral and/or practical guidance to the nursing assistants as needed.

##### Implementation of the intervention.

The intervention begins with an interview using the COPM [42, 43]. A researcher conducts the interview with the ward’s OT as an observer. The OT is present to understand the goal-setting process, as they will supervise the nursing assistants during the intervention. The OT will also contribute expertise related to participation in everyday life when formulating a measurable and realistic goal, within the relevant time frame.

During the interview, the older adult is asked to describe their daily life, including its activities and their performance, considering both the present and the past. The interview focuses on identifying both what works well and the challenges faced in daily life. Subsequently, the participant identifies and ranks the five most meaningful activities in their daily life. The participant also rates their occupational performance (COPM-P) and satisfaction (COPM-S) for each activity on a scale from 1 to 10.

In the second part of the interview, the older adult formulates a meaningful and relevant activity-based, person-centered goal for the intervention, based on one of the five prioritized activities identified in the previous step. The goal activity should be something the participant wants to perform or participate in but currently finds challenging or impossible. It must also be meaningful, relevant, and important for the participant’s well-being and QoL [42, 43]. An example of such a goal might be dressing independently, with the final goal being broken into smaller tasks that the participant practices with the nursing assistants during the 8 weeks of the Re-RAC intervention. Other examples might be moving independently to visit a neighbor in a different ward or participating in a social activity.

A goal-oriented support plan with specific activities to achieve the goal is developed by the OT together with the participant. A nursing assistant such as the participant’s contact person at the RAC can also be involved depending on the needs of the participant. These activities may include the use of assistive devices, environmental adjustments, and modified activity behaviors, such as physical exercise or task-specific training. Multiple training activities may be included and are tailored to the individual’s daily capacity. The time allocated for each participant is customized based on their needs and the nature of the goal. The intensity of the intervention can range from 2 to 7 days per week, with training sessions occurring once or multiple times per day, and each session lasting up to a maximum of 30 min.

The intervention lasts 8 weeks. The OT introduces the nursing assistants, who will support the older adult during the intervention, to the person-centered goal and the planned activities in the support plan. The nursing assistants receive supervision on how to support the specific participant according to the previously described support plan for staff. The intervention aims for ongoing progress with an increasing level of difficulty. Thus, the nursing assistants can adjust the level of difficulty based on the participant’s progress in their occupational performance. Additionally, the OT, PT, or the participants can initiate adjustments to improve the performance.

After 8 weeks, the intervention concludes with a renewed COPM assessment [42, 43], during which the participant’s occupational performance and satisfaction with the performance, related to the person-centered goal, are evaluated.

### Criteria for discontinuing or modifying allocated interventions {11b}

If a participant’s health condition deteriorates to the extent that they no longer meet the inclusion criteria, their participation in the study may be terminated by the researchers or by the OT in collaboration with the researchers.

The intervention is person-centered and tailored to each participant’s individual needs to achieve a personal and activity-based goal. It incorporates progression, in which the level of challenge in the training activities is gradually increased as the participant’s functional abilities improve. Consequently, the content and difficulty of the support plan are expected to be adjusted throughout the intervention. The criteria for these adjustments are based on the progression outlined in the goal-oriented support plan.

### Strategies to improve adherence to interventions {11c}

The goals of the intervention are person-centered, and the content of the intervention, including the activities designed to achieve the participant’s goal, is planned in collaboration with the participant. This collaborative approach is expected to enhance the participant’s motivation for and adherence to the intervention. Additionally, meetings with the OT after 3 and 6 weeks provide opportunities to discuss and adjust the support plan as needed, further supporting adherence. An information sheet will also be provided to participants, intended to be shared with relatives, to inform them about the study and potentially facilitate additional support from them. The support plan and the goal will also be visualized in each participant’s apartment.

To enhance the nursing assistants’ motivation and engagement, they will have access to support and supervision from the OT throughout the intervention period, as needed. Check-ups with the OT will be offered to both the older adult and the nursing assistants during weeks 3 and 6. The check-ups are intended to identify any need for changes in the goal-oriented support plan and to provide ongoing support for both the participant and the nursing assistants in progressing through the Re-RAC intervention. Additionally, the check-ups with the nursing assistants are also designed to help them maintain the participants’ motivation and engagement, thereby promoting adherence to the intervention.

To assess adherence to the intervention, all staff at the RAC facility are required to complete a logbook, as described later in this protocol. Adherence can also be partially monitored through the Medication and Care Support System (MCSS).

### Relevant concomitant care permitted or prohibited during the trial {11d}

RAC facilities included in the study must not concurrently participate in other rehabilitation-related research during the trial period. Re-RAC will be evaluated against TAU, which includes access to standard rehabilitation services when clinically indicated. Accordingly, participants in both the intervention and control groups may continue receiving ongoing rehabilitation programs—such as physiotherapy or occupational therapy—intended to maintain functional ability. These services will not be withdrawn or modified for control group participants and will proceed as per the conditions at the time of study inclusion. Adjustments will only be made if a new rehabilitation need arises that cannot be postponed.

Control group participants will set a goal but will not receive active support to achieve it during the trial period. Once this period concludes (after three months), the facility’s responsible occupational therapist will be informed, and the participant may then be offered Re-RAC outside the study framework.

For participants in the Re-RAC group, any existing rehabilitation services will be supplemented with actions outlined in the Re-RAC plan. Services in place at the time of inclusion may be modified if such adjustments better support the participant’s goal attainment during the intervention phase.

### Provisions for post-trial care {30}

No provisions for post-trial care are planned, given the expected low risk of harm associated with participation in the trial. Upon completion of the study, participants will continue with TAU based on medical indications and current guidelines. All participants are covered by patient insurance.

### Outcomes {12}

#### Primary outcome

The primary outcome is the mean change in occupational performance related to the prioritized activity, measured with COPM-P [42, 43], immediately after the 8-week intervention period. The comparison will be made between participants who have undergone the Re-RAC intervention and those receiving TAU.

#### Secondary outcomes

Secondary outcomes will compare mean changes from baseline to, 8 weeks from baseline, between older adults who have undergone the Re-RAC intervention and those receiving TAU in the following areas:

1. Self-perceived satisfaction with occupational performance in the prioritized activity, measured using the COPM-S [42, 43];
2. Self-perceived health-related quality of life, assessed with the EQ-5D-5L [44–46];
3. Self-perceived psychological wellbeing, measured with the WHO Well-Being Index (WHO-5) [47]; and.
4. Level of physical activity, assessed using an accelerometer (advanced pedometer) worn for seven consecutive days both before and immediately after the intervention; physical activity data will be supplemented with a diary documenting accelerometer wear time.

To explore any possible associations between pain and other outcomes, pain will be assessed using the pain dimension of the EQ-5D-5L.

Older adults’ experiences of the intervention will be investigated through individual semi-structured interviews conducted after completion of the intervention.

The experiences of staff involved in delivering the intervention will be investigated through focus-group interviews.

Finally, cost-effectiveness will be evaluated from a healthcare perspective by calculating the costs per quality-adjusted life year (QALY) gained.

### Participant timeline {13}

Table 1 and Fig. 1 summarize the schedule of enrollment, intervention, and data collection.

**Table 1:** Schedule of enrollment, interventions, and assessments

	**Study period**					
	**Enrollment**	**Baseline**		**Intervention**	**Post-intervention**	
**TIMEPOINT**	**w – 1 **	**w – 12 to w0**	**w0**	**w1 to w8**	**w9**	**w9 to w20**
**ENROLLMENT:**						
**Informed consent **	X					
**Eligibility screen**	X					
*MMSE-SR*	X					
*CFS-9*	X					
**Allocation**	X					
**INTERVENTIONS:**						
Intervention group				Re-RAC + TAU		
Control group				TAU		
**DATA COLLECTION:**						
*COPM*			X		X	
*EQ5D-5L*			X		X	
*WHO-5*			X		X	
*Accelerometers*			X		X	
*Interview participants*					X^a^	
*FGD nursing assistants*					X^b^	
*Medical records and administrative systems*		X				X

*CFS* Clinical Frailty Scale, *COPM* Canadian Occupational Performance Measure, *W* week, *FGD* focus group discussion, *MMSE-SR* Mini Mental State Examination—Swedish version, *TAU* treatment as usual, *Re-RAC* Reablement in Residential Aged Care

### Sample size {14}

A total of 86 individuals (*n* = 43 per group) will be included to detect an improvement of 0.9 units in the primary outcome, i.e., occupational performance, as measured using the Canadian Occupational Performance Measure—performance (COPM-P), between the intervention and control groups. This calculation assumes 80% power, a significance level of *α* = 0.05, and a two-sided *t*-test, with a standard deviation of 1.3 units for the change in COPM-P, and a 25% dropout rate. This corresponds to a moderate effect size (Cohen’s *d* = 0.5, with a baseline standard deviation of 1.8). Assumptions regarding the standard deviation, treatment effect, and dropout rate are based on findings from our previous feasibility study [28].

### Recruitment {15}

The managing director of all RAC facilities in the specific municipality will receive both written and oral information about the project and provide approval to initiate the study. Subsequently, the operation managers of the RAC facilities will receive oral information about the project and be asked to select three RAC facilities that meet the eligibility criteria for inclusion in the study. Ward managers at the selected RAC facilities will also receive information and participate in the staff training.

During a regular meeting for all residents in the somatic wards at the respective RAC facilities, members of the research team will provide verbal information about the project. Additionally, all potential residents will receive written information, including details about the Re-RAC intervention and study-specific aspects such as study goals, duration, participant roles, randomization, potential risks, voluntariness, and the written consent process. A few days after the information session, a research team member will visit each resident for an individual follow-up, address any questions, offer to read the information aloud if necessary, and ask whether the older adult wishes to participate. Those who express interest will provide written consent, after which eligibility screening will be conducted. We anticipate recruiting the participants over an 18-month period and the recruitment progress will be monitored monthly at research team meetings. If recruitment is slower than expected, contingency plans include extending the recruitment period or engaging additional facilities.

Nursing assistants who support participants in the intervention, along with rehabilitation staff and registered nurses at each participating ward, will receive both oral and written information during a ward meeting and be invited to participate as staff informants in focus-group interviews. Participating in the focus-group interviews is voluntary, but the staff are required to provide support for the intervention as part of their daily work.

## Assignment of interventions, allocation

### Sequence generation {16a}

Eligible participants will be randomized 1:1 to either treatment as usual (TAU) with Re-RAC as an add-on intervention or TAU alone, stratified by center (RAC facility). A computer-generated randomization sequence will be created using randomized permuted blocks of varying sizes.

### Concealment mechanism {16b}

To ensure allocation concealment, center-specific randomization lists will be prepared and distributed individually in sealed and sequentially numbered envelopes to the researcher responsible for the participant recruitment. These envelopes will be kept in a secure location and opened only after participant consent and screening have been completed.

### Implementation {16c}

The randomization sequence will be generated by the external statistician, who will not be involved in participant recruitment, enrollment, or assessment. A research team member will be responsible for enrolling participants and assigning them to interventions by opening the next envelope in sequence, following eligibility confirmation and consent.

## Assignment of intervention: blinding

### Who will be blinded {17a}

This study evaluates an intervention in which participants from both allocation groups may reside in the same ward, making it impossible to blind either the participant or the staff to their group assignment. Additionally, the nature of the intervention, which includes a training component delivered by the staff, precludes blinding for these groups. However, all researchers, except one, will remain blinded during the analyses. The unblinded researcher will be responsible for administering, collecting, and securely storing the coded data. This researcher will also be involved in the data collection and delivery of the intervention and therefore cannot be blinded to the randomization or the participants’ study IDs. Data entry into the electronic case report form (e-CRF) system called Greenlight Guru Clinical (GGC) will be performed by the unblinded researcher. Study ID numbers will remain concealed from the rest of the research team during the analyses. Outcome assessors and analysts, apart from the designated unblinded researcher, will remain blinded to group allocation. Statistical analyses will be conducted by an external, blinded statistician.

### Procedure for unblinding if needed {17b}

If any researcher is accidentally unblinded, they must not perform any tasks requiring blinding. Any instance of unblinding must be documented in a deviation log. All participant responses will be linked to a numerical pseudonym. In cases where unblinding becomes necessary, such as during an AE, the coding key may be accessed to identify the participant. 

## Data collection and management

### Plans for assessment and collection of outcomes {18a}

Following the eligibility screening, all participants will undergo the baseline assessment and wear an accelerometer for one week. The baseline assessment will be conducted using several questionnaires. To address potential challenges related to low vision and to reduce cognitive load, the questionnaires will be administered in an interview format. Additionally, the older adults will be advised to use their regular hearing and vision aids during all assessments, including MMSE-SR.

At the end of the intervention period, a follow-up assessment will be conducted, mirroring the baseline assessment. In conjunction with the follow-up, a proxy assessment of the older adults’ occupational performance (COPM-P) will be carried out by the OT at the RAC facility (Table 1).

To collect data on participants’ experiences, semi-structured individual interviews will be conducted with participants from the intervention group. Staff experiences will be collected through focus-group interviews after supporting at least one participant in the intervention.

Additionally, data on adherence, healthcare consumption, incidents, and medication use will be extracted from medical records and administrative systems for a period of 3 months before baseline and 3 months after the intervention concludes.

Any deviations from the study protocol or participant discontinuations will be documented.

### Measures

#### Canadian Occupational Performance Measure (COPM)

The COPM [43] is an interview-administered instrument used to assess occupational performance (COPM-P) and satisfaction with performance (COPM-S) for up to five self-determined activity issues in the areas of self-care, productivity, and leisure. Participants will rate their performance of and satisfaction with their highest-priority activity on a scale of 1–10, where 1 = unable to perform at all/not satisfied at all, and 10 = able to perform extremely well/extremely satisfied [42, 43].

The COPM has been used with individuals of various ages, diagnoses, and backgrounds and its psychometric properties are well established [43, 48, 49]. To validate the participants’ self-assessment of the performance (COPM-P), a proxy assessment will be conducted by an OT.

#### EQ-5D-5L

The EQ-5D-5L [44–46] is a widely used generic instrument for measuring health status. It consists of two parts: a descriptive system that assesses health across five dimensions (i.e., mobility, self-care, usual activities, pain/discomfort, and anxiety/depression), with each dimension having five response levels (i.e., no problems, slight problems, moderate problems, severe problems, and extreme problems/unable to). The second part is a visual analog scale (VAS), on which the participant rates their perceived health, ranging from 0 (the worst imaginable health) to 100 (the best imaginable health). In Re-RAC, the Swedish version of the interviewer-administrated version will be used. The EQ-5D-5L demonstrates strong psychometric properties across diverse populations, conditions, and settings [50].

#### World Health Organization Well-Being Index (WHO-5)

The WHO-5 is a widely used questionnaire designed to assess subjective psychological well-being. Each question is rated individually, with five statements evaluating how well they apply to the respondent over the past 14 days. Responses are scored on a scale from 5 (all the time) to 0 (none of the time), resulting in a total raw score ranging from 0 to 25 [47]. The questionnaire has demonstrated good internal and external validity when used with older adults [51]. In the Re-RAC study, the Swedish translation will be administered during a structured interview.

#### Accelerometer

A wrist-worn accelerometer with three axes (Actigraph GT3X +; ActiGraph, Pensacola, FL, USA) [52, 53] will be used to measure physical activity levels over two 7-day periods, one before and one after the intervention. Wrist-worn accelerometers have been shown to achieve high participant compliance [54]. Participants will also be provided with a diary covering the same time periods, in which to record the periods of time when the accelerometer is worn every day.

#### Participant experiences

Semi-structured individual interviews with 15–18 participants from the intervention group will be conducted to collect data on participant experiences. Participants from all participating RAC facilities will be asked to participate, and diversity in RAC facilities and participant characteristics will be striven for. The interviews will be conducted within 2 weeks following completion of the intervention. A study-specific interview guide will be used, focusing on participants’ experiences of the Re-RAC intervention, including their daily activities and perceptions of the professional team’s reception, support, and accessibility. Questions addressing the individual’s impact and participation in the intervention are also included. The interview guide was partially piloted during a feasibility study [28], with revisions made to improve the clarity of certain questions and terminology. Interviews will accommodate breaks as needed and will take place at a location chosen by each participant. A trained member of the research team will conduct the interviews, which will be audio-recorded and transcribed verbatim.

#### Staff experiences

Focus-group interviews will be conducted to capture staff experiences of Re-RAC. These experiences will be explored thorough six focus-group interviews (two at each RAC facility), with each group comprising five to eight staff informants [30]. A criterion-based inclusion approach will be employed, aiming for diversity of characteristics such as age, gender, education level, profession, and length of employment at the RAC facility. Recruitment of informants will continue until enough staff informants are available to form appropriately sized groups. A study-specific interview guide will be utilized, addressing experiences of the intervention in terms of its content, perceived benefits or drawbacks, reflections on the format and structure of the Re-RAC training, and how the intervention has influenced the staff members’ daily work. The interviews will be held in a meeting room at each RAC facility, and will be audio-recorded and transcribed verbatim. The same two moderators from the research group will conduct the interviews to ensure uniformity in facilitation. Before the first interview, the interview guide will be evaluated in terms of comprehensibility and relevance in a pilot interview.

#### Health-economic parameters

The health effect used in the health-economic evaluation will be measured in terms of QALYs. QALY gain will be calculated based on the EQ-5D-5L scores [44–46], comparing pre- and post-intervention data.

The cost estimates will include intervention costs and healthcare consumption costs. For the base case analysis, intervention costs will encompass all components of the intervention, including training, preparation, and execution. Costs for the training and preparation will be estimated based on information provided by the researcher responsible for the intervention. To monitor the extent to which each participant receives the intervention, and to capture adherence, all staff members will be required to complete a logbook located in each participant’s apartment every time they perform an intervention-related task, such as a training session with the participant. The logbook will include entries for the date, action/activity performed, time spent, and profession of the staff member who carried out the task. This information can also be partially monitored through the MCSS.

Healthcare resource utilization data will be retrieved from administrative systems covering primary care (e.g., NCS Cross or another relevant system), in- and outpatient care (e.g., InfoMedix PAS or equivalent), and municipal care (e.g., MCSS, TRESERVA, or similar systems) for the 3 months preceding and following the intervention period. Data on pharmacological prescriptions will be obtained from systems such as PASCAL, Läkemedel, or miniQ. All cost estimates will be costed using national Swedish tariffs. Medical records may also be consulted if necessary. The health-economic analysis is detailed in the health-economic analysis plan (HEAP), which is available from the researchers upon request.

#### Demographics

A study-specific questionnaire will be used to collect information on participants’ age, gender, country of birth, previous employment, educational background, current health status, and duration of residence at the study-specific RAC facility. These data will be collected at baseline only. Demographic information for the staff will be collected during the focus-group interview, including age, gender, education, and profession.

### Plans to promote participant retention and complete follow-up {18b}

Upon enrollment in the study, the research team will encourage participants to adhere to the intervention and complete the follow-up. However, it must be acknowledged that participants in this research project reside in RAC facilities and are, to some extent, frail. Their health status may deteriorate during the trial, potentially impacting retention rates in both groups. To support participant retention, flexible scheduling of interviews and data collection will be offered, including the option to divide follow-up interviews into shorter sessions to reduce fatigue. In addition, regular check-ups by rehabilitation staff will be provided to both participants and nursing assistants to maintain engagement and identify early signs of withdrawal risk. The research team will also work in close collaboration with RAC staff to facilitate reminders and logistical support. Clear and respectful communication will be emphasized throughout, highlighting the importance of each participant’s contribution to the study.

In the event that a participant discontinues the intervention or withdraws from the study, the research team will request permission to collect available follow-up data, if ethically and practically feasible. When provided, the reason for withdrawal will be documented. All data collected up to the point of withdrawal will be included in the final analysis, in accordance with the intention-to-treat principle.

### Data management {19}

A data management plan (DMP) has been created to describe how the research data will be collected, stored, handled, documented, used, and made available during and after the research project. The DMP can be obtained from the researchers upon request.

In summary, participants will be registered immediately after inclusion in the eCRF system (GGC) using their unique study IDs. During the assessments, all data will be entered into the GGC system by the non-blinded researcher. All paper forms will be labeled with the study ID and securely stored in accordance with local data management arrangements. Personally identifiable paper records, such as informed consent forms, will be stored separately from anonymized paper records.

Once data collection is complete, the database in GGC will be locked and exported to secure, encrypted, and backed-up servers. Any modifications to the raw data, as well as, all steps involved in the analysis, will be thoroughly documented. The storage of personal information and registration will comply with the European Union (EU) General Data Protection Regulation (GDPR).

All data will be retained for at least 17 years.

### Confidentiality {27}

In addition to what is described in the previous section regarding data management, only the research group will have access to the data during the data collection, analysis, and publication phases. An external statistician will have access to the data during the analysis phase. Personal ID numbers for the included participants will be delivered in encrypted form to the authorities responsible for medical records and administrative systems included in the study.

### Plans for collection, laboratory evaluation, and storage of biological specimens for genetic or molecular analysis in this trial/future use {33}

Not applicable, as no such collection will occur.

## Analyses

### Statistical methods for primary and secondary outcomes {20a}

Descriptive data will be presented as the mean and standard deviation (SD) or median and interquartile range (IQR) for numeric variables, as appropriate. Categorical variables will be presented as numbers and percentages. Baseline characteristics will be described by randomization group.

Efficacy analyses will be conducted according to the intention-to-treat (ITT) principle, analyzing participants according to their original randomization assignment. Primary and secondary outcomes will be compared between groups using analysis of covariance (ANCOVA), adjusted for baseline values, and study center. All instrument scales (e.g., COPM, EQ-5D-5L, WHO-5) will be treated as continuous variables in the analyses. Robust standard errors (using the HC3 method) will be employed to ensure valid inferences in the presence of deviations from normality. Results will be presented as adjusted mean differences between groups, with corresponding 95% confidence intervals (CIs). The number and proportion of participants showing improvements of more than 1, 2, or 3 units will also be presented descriptively and compared between groups. The level of physical activity will be classified as sedentary, light intensity, or moderate to vigorous as per Hansen et al. and Forster et al. [55, 56].

All tests will be two-tailed and conducted at a 5% significance level. Missing data will be handled using multiple imputation. Statistical analyses will be performed using SAS/STAT® Software, Version 9.4 or later (SAS Institute Inc., Cary, NC, USA) and IBM SPSS Statistics 26 or later (IBM Corp., Armonk, NY, USA).

### Analysis of qualitative data

The individual semi-structured interviews will be analyzed using qualitative thematic analysis [29].

The analysis process will follow six steps [57]. Step 1 begins with familiarization with the material by reading and listening to the interview data. In Step 2, codes will be generated, followed by Step 3, in which the search for themes will begin. In Step 4, the themes will be reviewed, and in Step 5, they will be finalized and named. Finally, in Step 6, the manuscript will be written.

In the focus-group interviews with nursing staff, data will be analyzed using the methods described by Krueger and Casey [30] and Dahlin-Ivanoff and Holmgren [31]. The analysis will generate categories that reflect the discussions of the focus group participants relevant to the purpose of the project, emphasizing the meaning derived from the collective discussions rather than individual comments [30].

To strengthen the credibility of the analysis process, all researchers involved in the project will to some degree participate in the analysis. The process will continue until consensus is reached among the researchers.

All data will be analyzed using NVivo (QSR International, Inc., Cambridge, MA, USA) qualitative data analysis software.

### Health-economic analysis

A cost–utility analysis using individual data will be performed from a healthcare perspective [58]. Cost-effectiveness ratios will be based on the changes in QALY and net costs for the intervention group compared with the control group. Results will be presented as an incremental cost-effectiveness ratio (ICER), expressed as ICER = Ca − Cb/Ea − Eb, where Ca and Cb represent the costs for the intervention and control groups, respectively, and Ea and Eb represent the QALY changes for the intervention and control groups, respectively. Sensitivity analyses will be conducted to account for uncertainty in parameter estimates by incorporating both British [59] and Swedish [60] value sets for the health state preferences, as well as varying costing approaches.

QALYs will be derived from the EQ-5D-5L [44–46] using the summary index score based on societal preference weights (“utilities”). The health state index scores range from less than 0 (a health state equivalent to dead, with negative values representing values worse than dead) to 1 (the value of full health). Health state preferences typically represent national or regional values and can therefore vary among countries or regions. In this study, both the widely used British value set [59] and the recently developed Swedish value set [60] will be applied.

### Interim analyses {21b}

No interim analysis will be performed.

### Methods for additional analyses (e.g., subgroup analyses) {20b}

There are no predefined subgroup analyses. Exploratory analyses, including subgroup analyses and interaction analyses, may be performed at a later stage. All significant findings from these exploratory analyses will be considered hypothesis-generating and will require confirmation in future studies.

### Methods in analysis to handle protocol non-adherence and any statistical methods to handle missing data {20c}

Protocol non-adherence will be managed according to the ITT principle, analyzing all randomized participants according to their original randomization assignment, regardless of withdrawal, protocol deviations, or noncompliance. Missing values within each instrument will be handled according to the instructions provided in the respective instrument manuals, if available. Other missing data will be addressed using multiple imputation by chained equations.

### Plans to give access to the full protocol, participant-level data, and statistical code {31c}

The datasets generated and/or analyzed during the current study will not be made publicly available due current Swedish ethical legislation and the EU GDPR. However, data may be available from the PI on reasonable request, provided that appropriate permissions are obtained from the relevant authorities.

## Oversight and monitoring

### Composition of the coordination center and trial steering committee {5d}

This research project originates from collaboration among Region Örebro County, Örebro Municipality, and Örebro University, with the aim of increasing the research engagement in the municipality. The project is coordinated by a research group established at the University Health Care Research Center within Region Örebro County. The research group, which also includes researchers from Kristianstad and Mälardalen universities, has ultimate responsibility for the trial, trial design, and adherence to the study protocol.

The PI will ensure that the trial is conducted in line with good clinical practice, oversee the trial, manage trial registration, oversee the DMP, and ensure overall quality. One member of the research group will coordinate communication with the municipality, management, and RAC facilities, and will also be responsible for the intervention, including training and maintaining contact with the staff. Another research group member will be responsible for the HEAP, while an external statistician will be responsible for the statistical analysis plan (SAP). The research team will meet monthly.

A formal Trial Steering Committee (TSC) will not be established. However, strategic oversight and major decisions will be reviewed and approved by a steering group formed through the collaboration among Region Örebro County, Örebro Municipality, and Örebro University (The Collaboration Forum for older adults, Forum 2.0), in addition to the regular meetings of the core research group. The progress of the research project is reported to this steering group (Forum 2.0) every 2 years. During these meetings, independent researchers review the study’s progress to ensure it is proceeding as planned.

### Composition of the data monitoring committee, its role and reporting structure {21a}

The steering group formed through the collaboration among Region Örebro County, Örebro Municipality, and Örebro University (The Collaboration Forum for older adults, Forum 2.0) will also maintain overall oversight of the project as no formal Data Monitoring Committee (DMC) will be established. Additionally, Region Örebro County will be responsible for addressing data safety and quality issues, and for safeguarding the interests of the study participants.

### Adverse event reporting and harms {22}

AEs are defined as; any untoward medical occurrence in a study participant, regardless of its relationship to the intervention. A serious AE (SAE) are defined as an AE that results in death, is life-threatening, requires inpatient hospitalization or prolongation of existing hospitalization, or results in persistent or significant disability or incapacity.

Any AE or harms resulting from study participation will be reported and managed by the on-site clinical staff or the research team, depending on the nature of the event. Management will follow standard clinical care procedures.

AEs will be collected throughout the study period using both unsolicited reporting (initiated by participant or staff) and solicited reporting through direct questioning during follow-up interviews and other participant interactions.

All AEs and SAEs will be documented in the medical records and recorded using a standardized e-CRF to ensure consistent assessment of severity, based on predefined criteria outlined in the Common Terminology Criteria for Adverse Events (CTCAE). Collected data will include onset, duration, severity, relationship to the intervention, expectedness, and outcome. Events will be evaluated by unblinded research staff in collaboration with healthcare professionals.

Severity will be graded on a scale from 1 to 5 (mild, moderate, severe, life-threatening, and death). Each AE will also be assessed for its likelihood of being related to the intervention, and whether it is expected or unexpected based on known or anticipated risks, including differences in nature, severity, or frequency.

SAEs will be reported to the PI within 24 h of becoming known. Non-serious AEs will be documented and reviewed during regular project meetings and included in interim reports. If required, external reporting to oversight bodies (e.g., ethics committee or sponsor) will be conducted in accordance with national and institutional guidelines.

Participants experiencing AEs will be referred to their usual healthcare providers. All AEs will be monitored until resolution or stabilization.

Potential unintended effects related to the trial process itself (e.g., stress from assessments or burden from participation) will be monitored thorough participant feedback, staff reports, and observed behavior. Any such findings will be reviewed and addressed by the core research team in collaboration with the responsible rehabilitation staff.

### Frequency and plans for auditing trial conduct {23}

Given the nature and scope of the trial — a pragmatic, non-pharmacological intervention conducted within existing RAC facility structures — a formal independent auditing process was not deemed necessary or feasible. Oversight will instead be maintained through regular internal monitoring by the core research team, ongoing collaboration with RAC facility staff, and periodic reporting to the Collaboration Forum for Older Adults (Forum 2.0), which includes independent researchers who review study progress.

### Plans for communicating important protocol amendments to relevant parties (e.g. trial participants and ethical committees) {25}

Any modifications of the protocol will be posted in the trial registry and communicated to the ethical board and other relevant parties.

### Dissemination plans {31a}

The results of this study will be presented at relevant national and international conferences and published in peer-reviewed journals. Both positive and negative results will be reported. Dissemination of the findings will also include written and oral presentations to municipalities and the research community. Additionally, the findings will be communicated, either orally or in writing, to the participants and participating wards.

## Discussion

Previous research on reablement interventions for older adults has primarily focused on individuals in ordinary housing, highlighting a growing need for interventions in RAC facilities, where the reablement approach shows promise but needs further evaluation [20]. This study is a PrRCT designed to evaluate the efficacy, efficiency, and experiences of a reablement intervention, Re-RAC, tailored to older adults residing in RAC facilities. The findings of this study will provide valuable knowledge to guide the future implementation and evaluation of reablement interventions in the RAC context.

The Re-RAC intervention will be evaluated in a real-world context, i.e., in RAC facilities and involving both older adults residing there and the staff. Conducting research in a real-world setting has certain advantages, such as implementing the intervention at the same time as it is being researched, and conducting the evaluation in the same context in which it is intended to be used, with all its complexity. However, there are also potential risks that need to be taken into consideration, such as procedural drift, a common problem in studies evaluating complex health interventions in a real-world setting [61]. Awareness of this potential risk is important in this PrRCT, especially in view of how hard it can be to change established and habitual working practices [62], as confirmed by staff participating in our feasibility study who asked for more supervision [28]. To prevent procedural drift in this study, the staff training will be very clear as to how Re-RAC differs from TAU, difficulties will be acknowledged, guidance in how to implement the new practice will be supplied, and extended supervision will be offered. Furthermore, it is important to establish a good working relationship between the researchers and the teams at the participating RAC facilities, to create a positive attitude toward changing behavior in both parties [61].

The intervention adopts a person-centered approach, with activity-based goals selected by the participants themselves, making the process more engaging and understandable, potentially enhancing confidence in their abilities. Previous studies have shown that when older adults do not fully understand the aims and process of reablement, their engagement and participation are diminished [24]. Therefore, the intervention’s person-centered and tailored approach to each participant’s individual needs and activity-based goals, represents a key strength of this planned PrRCT.

The Re-RAC study was preceded by a feasibility study that confirmed that the intervention and study procedure were feasible [28]. Adjustments were made, particularly regarding the training and support provided to the nursing assistants. Regular check-ups will be introduced to help staff maintain participants’ motivation and engagement, provide ongoing support, and identify necessary adjustments to support plans. In earlier reablement intervention studies, staff training has often been inadequately detailed, making it difficult to distinguish differences in the implementation of reablement across studies, and complicating comparisons [20]. Therefore, efforts have been made to clearly describe both the staff training and the Re-RAC intervention to ensure transparency and rigor in the study, as well as to enhance its comparability to other studies.

The present study aims to recruit 86 participants to detect a moderate-sized treatment effect. Clinical studies often encounter several challenges, including the quality of the outcome measures used, adherence to the intervention, and data collection [63]. In the planned PrRCT, these challenges may be amplified due to the frailty of the older adult participants. Data collection in such a population is inherently challenging, as seen in earlier studies [64, 65]. To address this, this study has limited the number of assessments and outcome measures and will conduct the assessments as structured interviews. This approach was tested in the feasibility study, which demonstrated that neither the participants nor the staff found the data collection process overly demanding [28]. However, it may be difficult to reach the estimated sample size in the included RAC facilities, so we have the municipality’s approval to include additional RAC facilities if needed. Furthermore, to gain a comprehensive understanding of the intervention’s impact on the older adults, nursing staff, and overall healthcare, the intervention will be evaluated using quantitative, qualitative, and health-economic methods. These diverse methods provide multiple perspectives, thereby enriching the findings.

## Trial status

This is the first version of the study protocol, dated May 2025. A feasibility study for the trial was conducted in 2023 [28]. The ethical application was approved by the Swedish Ethical Review Authority (Dnr 2024–01474-01) in April 2024 with an amendment approved in December 2024 (Dnr 2024–07409-02). Staff training was carried out from September 2024 to January 2025. Recruitment of study participants began in October 2024 and is expected to continue until the estimated number of participants has been reached, likely taking at least 18 months. The data collection process will be temporarily paused during the summer months of both 2025 and 2026, in alignment with the regular vacation period of the staff, which spans June, July, and August. Baseline data started in October 2024, and follow-ups began in November/December 2024. Recruitment and data collection are anticipated to be completed by 31 December 2026.

## Data Availability

The datasets from the trial will be stored for 17 years. The datasets generated and/or analyzed during the current study will not be made publicly available due current Swedish ethical legislation and the EU GDPR. However, data may be available from the PI on reasonable request, provided that appropriate permissions are obtained from the relevant authorities. Information material, training resources, data, and program codes will be available upon reasonable request.

## Abbreviations

AE: Adverse event
ANCOVA: Analysis of covariance
CFS-9: Clinical Frailty Scale-9
CHEERS: Consolidated Health Economic Evaluation Reporting Standards
CI: Confidence interval
CTCAE: Common Terminology Criteria for Adverse Events
CONSORT: Consolidated Standards of Reporting Trials
COPM: Canadian Occupational Performance Measure
COPM-P: Canadian Occupational Performance Measure—performance
COPM-S: Canadian Occupational Performance Measure—satisfaction
COREQ: Consolidated Criteria for Reporting Qualitative Research
CMOPE: Canadian Model of Occupational Performance and Engagement
DMP: Data management plan
eCRF: Electronic case report form
EU: European Union
GDPR: General Data Protection Regulation
GGC: Greenlight Guru Clinical
HEAP: Health-economic analysis plan
ICER: Incremental cost-effectiveness ratio
IQR: Inter-quartile range
ITT: Intention to treat
MCSS: Medication and Care Support System
MMSE-SR: Mini Mental State Examination—Swedish version
OT: Occupational therapist
PrRCT: Pragmatic randomized controlled trial
PT: Physiotherapist
QALY: Quality-adjusted life year
QoL: Quality of life
RAC: Residential aged care
Re-RAC: Reablement Intervention in Residential Aged Care
RCT: Randomized controlled trial
SAE: Serious adverse event
SAP: Statistical analysis plan
SD: Standard deviation
SPIRIT: Standard Protocol Items: Recommendations for Interventional Trials
TAU: Treatment as usual
TIDieR: Template for Intervention Description and Replication
TSC: Trial Steering Committee
VAS: Visual analog scale
WHO: World Health Association
WHO-5: WHO Well-Being Index
